# Chronic eosinophilic pneumonia: a case report and review of the literature

**DOI:** 10.4076/1757-1626-2-7735

**Published:** 2009-07-02

**Authors:** Soichi Sano, Keiko Yamagami, Katsunobu Yoshioka

**Affiliations:** Department of Internal Medicine, Osaka City General HospitalOsakaJapan

## Abstract

A 66-year-old female patient was admitted with a one year history of low-grade fever and shortness of breath with worsening symptoms. A computed tomography (CT) scan of the chest was performed, which revealed bilateral homogenous consolidation with subpleural predominance in the corresponding area. The percentage of eosinophils in the bronchoalveolar lavage (BAL) fluid was 98% and histological examination showed remarkable accumulation of eosinophils and lymphocytes in the alveoli and interstitium, with mild interstitial fibrosis. The diagnosis of idiopathic chronic eosinophilic pneumonia was confirmed. Intravenous methylprednisolone pulse therapy (500 mg daily) for three days followed by 30 mg of oral prednisolone showed dramatic response. She was essentially normal after one year follow up.

CEP is characterized by chronic and progressive clinical features and specific pathological findings. We review the epidemiology, clinical, diagnosis, and therapy of the CEP.

## Case presentation

A 66-year-old Japanese female was admitted with a one year history of low-grade fever and shortness of breath with worsening symptoms. Patient details were as follows: Occupation: housewife; Ethnicity: Japanese; Weight: 46.9 Kg; Height: 152.5 cm; Medical history: 1 year history of asthma, otherwise unremarkable; Family history: unremarkable; Patient habits and medications: non-smoker, no alcohol consumption, current medications of fluticasone propionate/salmeterol xinafoate inhalation, tulobuterol adhesion patch.

On examination her vital signs were stable, she was afebrile and her oxgen saturation was 94% on ambient air. She appeared tired but not in any distress. Auscultation of the lungs revealed wheeze bilaterally and coarse crackles at the right middle and the left base. The remainder of her examination was unremarkable. Her white blood cell count was 18840/l with eosinophils of 62.3%. The IgE level was 1791 IU/ml. A comprehensive metabolic profile was normal and urine analysis did not show any sediment. Arterial blood gas analysis included pH 7.432, PCO_2_ 36.5 mmHg, PO_2_ 60.7 mmHg, and HCO3^−^ 23.9 mmol/l. Her chest radiograph showed infiltration in the right middle, left upper, and left lower field. A computed tomography (CT) scan of the chest was performed, which revealed bilateral homogenous consolidation with subpleural predominance in the corresponding area. The percentage of eosinophils in the BAL fluid was 98% and histological examination showed remarkable accumulation of eosinophils and lymphocytes in the alveoli and interstitium, with mild interstitial fibrosis. Based on these observations, the diagnosis of idiopathic chronic eosinophilic pneumonia was confirmed.

Intravenous methylprednisolone pulse therapy (500 mg daily) for three days followed by 30 mg of oral prednisolone was started. Patient showed dramatic response to the treatment and discharged on day 27. A dosage of predonisolone was gradually tapered without recurrence of CEP for one year.

## Discussion

The term eosinophilic lung disease describes a group of entities characterized by accumulation of eosinophils in the pulmonary interstitium and airspaces. They could be classified as follows; Eosinophilic lung disease of unknown cause (simple pulmonary eosinophilia (SPE) Loffler’s syndrome), acute eosinophilic pneumonia (AEP), chronic eosinophilic pneumonia (CEP), idiopathic hypereosinophilic syndrome (HES), ii. Eosinophilic lung disease of known cause (allergic bronchopulmonary aspergillosis (ABPA), bronchocentric granulomatosis (BG), parasitic infections, drug reactions), iii. Eosinophilic vasculitis (allergic angiitis, Churg-Strauss syndrome) [1]. Although peripheral eosinophilia is common feature of these diseases, it could be absent.

CEP was first described by Carrington CB in 1969 as chronic variants of Loffler’s syndrome [2]. The clinical picture of the disease is progressive and severe illness characterized by high fever, weight loss, night sweats, and shortness of breath. Most patients are middle aged and women are more frequently affected than men. Because approximately 50% of the patient has the past history of asthma, Churg-Strauss disease is one of the top differential diagnosis. Symptom is often severe, but life-threatening respiratory dysfunction is not common [2].

The typical chest radiographic finding in CEP is nonsegmental infiltration with peripheral predominance (so called “photographic negative of pulmonary edema”) involving mainly the upper lobe. However, this finding could be seen in only 50% of the patients. High-resolution CT scan demonstrates (a) patchy consolidation with peripheral and upper lobe predominance (most common finding in CEP), (b) ground-glass opacities with crazy paving, (c) bandlike subpleural opacities. These findings could be seen in Loeffler’s syndrome. Loeffler’s syndrome, however, is self-limiting disease and the areas of consolidation disappear within several days [3].

The integration of these characteristic clinical and radiological findings could obviate the pathological examination in most case of CEP. In some cases, however, require the pathologic findings for confirming the diagnosis because there are diagnostic pitfalls due to some overlapping features among eosinophilic lung diseases.

There are some patients who present with minimal blood eosinophilia and in whom peripheral distribution of infiltrate is not apparent. In such cases, Bronchoalveolar lavage can be useful in the diagnosis of eosinophilic lung diseases because normal BAL fluid consists of less than 1% of eosinophils. A finding of 20% of more eosinophils is almost always associated with eosinophilic alveolitis. The histological findings of CEP include (a) interstitial and intra-alveolar exudates of eosinophils (more than 2/3 of the patients), (b) bronchiolitis obliterans (25%), (c) eosinophilic microabscesses, focal areas of intra-alveolar necrosis, sarcoid-like granulomas, Charcot-Leyden crystals, organizing pneumonia (less common) [4].

The prognosis of CEP is excellent. Although clinical manifestation reveals protracted course without therapy, CEP should prompt the physicians for oral administration of corticosteroids. CEP is promptly responsible for oral administration of corticosteroids. Because short-term relapse is observed in 80% patients, prolonged therapy is required for the management of CEP [4].

**Figure 1.**
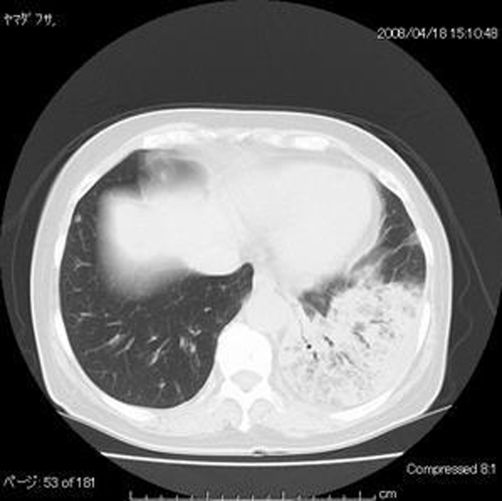
Thin-section CT scan (lung windowing) shows homogenous consolidation with subpleural predominance in the left lower lung.

**Figure 2.**
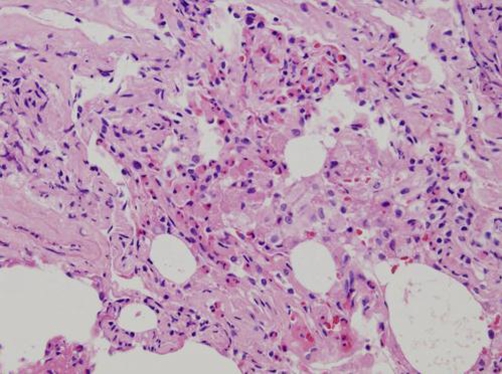
Histological examination showed remarkable accumulation of eosinophils and lymphocytes in the alveoli and interstitium, with mild interstitial fibrosis.

## Conclusion

CEP is characterized by chronic and progressive clinical features and specific pathological findings. We review the epidemiology, clinical, diagnosis, and therapy of the CEP.

## Abbreviations

BAL: bronchoalveolar lavage
CEP: chronic eosinophilic pneumonia
